# Characterization of a novel STAT 2 knock-out hamster model of Crimean-Congo hemorrhagic fever virus pathogenesis

**DOI:** 10.1038/s41598-020-69054-3

**Published:** 2020-07-23

**Authors:** Charlene Ranadheera, Emelissa J. Valcourt, Bryce M. Warner, Guillaume Poliquin, Kyle Rosenke, Kathy Frost, Kevin Tierney, Greg Saturday, Jinxin Miao, Jonna B. Westover, Brian B. Gowen, Stephanie Booth, Heinz Feldmann, Zhongde Wang, David Safronetz

**Affiliations:** 1 Zoonotic Diseases and Special Pathogens, National Microbiology Laboratories, Public Health Agency of Canada, Winnipeg, MB Canada; 2 Department of Medical Microbiology, University of Manitoba, Winnipeg, MB Canada; 3 Office of the Scientific Director, National Microbiology Laboratories, Public Health Agency of Canada, Winnipeg, MB Canada; 4 Department of Pediatrics and Child Health, University of Manitoba, Winnipeg, MB Canada; 5 Laboratory of Virology, Division of Intramural Research, National Institute of Allergy and Infectious Diseases, National Institutes of Health, Rocky Mountain Laboratories, Hamilton, MT USA; 6 Rocky Mountain Veterinary Branch, National Institute of Allergy and Infectious Diseases, National Institutes of Health, Rocky Mountain Laboratories, Hamilton, MT USA; 7 Department of Animal, Dairy, and Veterinary Sciences, Utah State University, Logan, UT USA; 8 Department of Pathology, School of Basic Medical Sciences, Zhengzhou University, Zhengzhou, 450066 People’s Republic of China; 9 Present Address: Bioforensics Assay Development and Diagnostics, National Microbiology Laboratory, Public Health Agency of Canada, Winnipeg, MB Canada

**Keywords:** Infection, Pathogenesis, Diseases, Infectious diseases, Viral infection

## Abstract

Crimean-Congo hemorrhagic fever virus (CCHFV) is a tick-borne pathogen causing a febrile illness in humans, which can progress to hemorrhagic manifestations, multi-organ failure, and death. Current mouse models of CCHFV infection reliably succumb to virus challenge but vary in their ability to reflect signs of disease similar to humans. In this study, we established a signal transducer and activator of transcription 2 (*STAT*2) knockout hamster model to expand the repertoire of animal models of CCHFV pathogenesis that can be used for therapeutic development. These hamsters demonstrated a systemic and lethal disease in response to infection. Hallmarks of human disease were observed including petechial rash, blood coagulation dysfunction, and various biochemistry and blood cell count abnormalities. Furthermore, we also demonstrated the utility of this model for anti-CCHFV therapeutic evaluation. The STAT2 knock-out hamster model of CCHFV infection may provide some further insights into clinical disease, viral pathogenesis, and pave the way for testing of potential drug and vaccine candidates.

## Introduction

Crimean-Congo hemorrhagic fever virus (CCHFV) is a segmented, negative-sense RNA virus belonging to the *Orthonairovirus* genus within the *Nairoviridae* family. Endemic in Africa, Southeast Europe, the Middle East and Asia, CCHFV causes a severe and sometimes fatal disease in humans with a case fatality rate ranging from 3–80%^1–8^. CCHFV is transmitted from *Hyalomma* ticks to humans, wildlife, and livestock^9^. Transmission can also occur through direct contact with fluids or tissues of infected livestock or patients and can lead to nosocomial outbreaks of CCHFV in the hospital setting. Disease in humans begins as a febrile illness including fever, chills, headache, and muscle soreness, which can either resolve or progress to a hemorrhagic state. The hemorrhagic state includes petechiae, liver necrosis, hemorrhaging of internal organs, shock, and death can occur from multi-organ failure^10–15^. Neurological illness associated with CCHFV infection has also been documented^14–18^. Laboratory findings associated with mortality in humans include elevated liver enzymes, thrombocytopenia, intravascular coagulopathy, and cytokine responses^5,16,19–29^. Though there are no licensed therapeutics to treat CCHFV infection, ribavirin, a nucleoside analog, has demonstrated efficacy against several viruses and has been proposed as a possible treatment. However, its efficacy against CCHFV in humans continues to be the subject of much debate^11–13^.

To improve therapeutic development for CCHFV, animal models of CCHFV pathogenesis used for pre-clinical testing should ideally exhibit both similar clinical signs of human disease and similar laboratory findings associated with mortality in humans. Cynomolgus macaques are the only immunocompetent animal model demonstrating both of these characteristics^30^. However, the non-human primate model for CCHFV faces limitations for evaluation of medical countermeasures because it is not consistently lethal and is associated with increased cost and limited availability. Adult immunocompromised mice have been established as a cost-effective and practical model for the study of CCHFV pathogenesis and drug evaluation. Signal transducer and activator of transcription (*STAT*) 1 gene knockout (*STAT1*^*−/−*^) mice ^31^, IFN α/β receptor gene knockout (*IFNAR*^*−/−*^) mice^31–34^, adult wild-type mice subject to antibody-mediated disruption of type I IFN signalling^35^, and humanized mice expressing human immune cells (Hu-NSG-SGM3)^36^ have been established as lethal mouse models of CCHFV infection. While these small animal models have been useful for further understanding CCHFV pathogenesis, they vary in their ability to exhibit similar clinical signs of human disease and similar laboratory findings associated with mortality in humans.

Recently, golden Syrian hamsters (*Mesocricetus auratus*) devoid of functional *STAT2* (*STAT2*^*−/−*^) have demonstrated success in modeling viral infection and have been used to highlight the importance of the type I IFN response in viral control of human adenovirus ^37–40^. The S*TAT*2 gene deletion in this model was shown to impair type I interferon signalling, impairing gene transcription induced by IFN-α and IFN-β, delaying production of IFNα transcripts, and decreasing the production of MHC-1 transcripts. However, the generation of an antibody and T-cell response were seemingly unchanged^38^. Furthermore, there were no observed deficiencies in IFN-γ and IL-4 transcripts^38^. We aimed to expand the repertoire of small animal models available for the study of CCHFV pathogenesis and pre-clinical testing of therapeutics by characterizing the progression of CCHFV-associated disease in a lethal STAT2^*−/−*^ hamster model.

## Methods

### Ethics and biosafety

All animal work was approved by the Animal Care Committee of the Canadian Science Centre for Human and Animal Health following the guidelines of the Canadian Council for Animal Care. All work with infectious material was conducted in the Biocontainment Level 4 (BSL-4) suite at the National Microbiology Laboratory (Winnipeg, Canada) following approved standard operating procedures.

### Cells, virus, antivirals, and animals

Human adrenal gland/cortex SW13 cells (ATCC, VA, USA) were cultured in L15 media (Leibovitz’s L15 media, ThermoFisher Scientific, ON, Canada) with 10% fetal bovine serum (FBS). Cells were incubated in H_2_O-saturated atmosphere conditions at 37 °C with no CO_2_. The strain IbAr10200 of CCHFV (Accession No: NC_005300.2, NC_005300.1, NC_005302.3) was a kind gift from the University of Texas Medical Branch (Galveston, Texas). IbAr10200 was cultured in SW13 cells using L15 media supplemented with 1% FBS. Commercial ribavirin (R&D Systems, Minneapolis, MN) was dissolved in PBS for treatment of hamsters. STAT2^*−/−*^ hamsters were developed and bred at the Department of Animal, Dairy, and Veterinary Sciences at Utah State University^41^.

### Hamster infections and treatment

Groups of both male and female *STAT2*^*−/−*^ hamsters aged 6–8 weeks were provided by Dr. Zhongde Wang from the Department of Animal, Dairy, and Veterinary Sciences at Utah State University. The *STAT2*^*−/−*^ hamster strain comprises F2 homozygotes derived from an LVG golden Syrian hamster female genetically modified by CRISPR/Cas9-mediated mutation of the STAT2 N-terminal domain^41^. On 0 days post-infection (dpi), hamsters were anesthetized by inhalation of isoflurane and virus was delivered by intraperitoneal, intramuscular, or subcutaneous routes of inoculation in 100 µl, 2 × 50 µl, or 200 μl of L15 media, respectively. Clinical signs and weights were recorded daily. On sacrifice days, hamsters were anesthetized by inhalation of isoflurane, bled by cardiac puncture and euthanized by anesthetic overdose with isoflurane. Blood was divided for blood chemistries, total cell blood counts, coagulation assays, cytokine profiling, and antibody detection. Tissues were harvested and fixed in 10% neutral buffered formalin for pathology or homogenized in L15 media and clarified by centrifugation for viral titration on SW13 cells. A set of animals (n = 9) were treated with 100 mg/kg/day of Ribavirin (100 μl) beginning on 1 dpi or 2 dpi. Hamsters were treated with 100 mg/kg/day of Ribavirin for two days and then the dose of Ribavirin was dropped to 75 mg/kg/day for a further 12 days. Trial bleeds were conducted on treated hamsters on 3 dpi and 6 dpi to test for the presence of virus. A set of three animals were humanely euthanized at 8 dpi and serum was collected. Animals were euthanized when they reached humane endpoints, defined as exhibiting multiple of the following signs: Abnormal swelling, protrusion (hernia, rupture), abnormal discharge, severe dehydration, twitching, tremors, convulsions, paralysis, unsteady gait, lameness, muscle flaccidity, rigidity or weakness. Immediate euthanasia was conducted for animals that demonstrated either > 20% weight loss or unresponsiveness. The remaining survivors at the termination of the study (26 dpi) were humanely euthanized and terminally bled for serum collection.

### Viral titrations

SW13 cells were seeded onto 96-well dishes one day prior to infection. Blood, heart, lung, liver, spleen, and kidney tissues were homogenized in 500 μl of L15/1% FBS using a TissueLyserII at a frequency of 30 for 6 min (QIAGEN, Limburg, Netherlands). The homogenates were clarified by centrifugation at 10,000 g for 10 min and supernatants were serially diluted in L15/1% FBS. 100 μl of each virus dilution was added to the cells in each well. The presence of viral-induced cytopathic effect (CPE) was read at 7 dpi by the appearance of rounded cells and/or destruction of the monolayer and TCID_50_ values were calculated according to Spearman-Karber method^42^. Each biological sample was tested in triplicate.

### Histopathologic and immunohistochemistry analysis

For histopathological studies, lung, kidney, and heart samples were fixed in 10% neutral buffered formalin, embedded in paraffin, sectioned at 5 µm and stained with hematoxylin and eosin for examination on the Mirax Midi imaging system. For immunohistochemistry, paraffin tissue sections were rehydrated through a series of xylene and decreasing ethanol concentrations. Antigen retrieval was then performed in Biocare’s Decloaking Chamber using 10 mM Na Citrate 6.0 pH for 15 min at 110 °C. After cooling, protocol was followed according to the manufactures instructions for the Anti-Mouse HRP Cell and Tissue IHC Staining Kit (R&D Systems, CTS002). Slides were blocked both with the peroxidase blocking reagent and serum blocking reagent followed by an avidin block. All rinses were performed with 1X TBS-Tween. The slides were then incubated overnight in a 1:200 dilution of a mouse monoclonal antibody specific for CCHFV nucleoprotein (N) (NR-40257, a gift from BEI resources). Slides were then rinsed again with 1X TBS-Tween and then incubated with the biotinylated secondary antibody supplied by the kit for 30 min, followed by incubation of HSS-HRP for 30 min. DAB was then applied for two minutes and then rinsed with distilled water. Slides were then counterstained with hematoxylin 7,211 (Richard Allen Scientific), dehydrated through a series of increasing ethanol concentrations to xylene and then cover-slipped with Permount mounting medium.

### Hematologic, coagulation, and blood chemistry

Complete blood counts were obtained using a VetScan HM5 (Abaxis Veterinary Diagnostics) as per manufacturer’s instructions and serum biochemistry was analyzed using a VetScan VS2 (Abaxis Veterinary Diagnostics) using comprehensive profile discs in accordance with manufacturer’s instructions. To measure coagulation parameters a Start4 instrument (Diagnostica Stago) was used according to manufacturer’s instructions.

### Immunoassays

To quantify serum IgG raised in STAT2^*−/−*^ hamsters infected with CCHFV, anti-CCHFV nucleoprotein IgG ELISAs were conducted. Briefly, 96-well half area flat-bottom high-binding microplates (Corning, New York, USA) were coated with recombinant CCHFV nucleoprotein diluted in sodium bicarbonate (50 mM) at a concentration of 1.25 µg/mL and incubated at 4 °C overnight. Wells were washed using PBS-T (PBS, 0.05% Tween-20) and blocked in diluent (PBS, 5% milk, 0.5% Tween-20) at room temperature for 1 h. Serum from STAT2^*−/−*^ hamsters was two-fold serially diluted in diluent. Diluted samples were added to corresponding wells of the coated ELISA plates and incubated at 37 °C for 1 h. Plates were washed six times with PBS-T. Goat anti-hamster IgG horse radish peroxidase-conjugated antibody (KPL Inc., Gaithersburg, USA) diluted 1 in 10,000 was added to each well of the microplate. The plates were allowed to incubate at 37 °C for 1 h. Plates were washed six times with PBS-T. ABTS peroxidase substrate (KPL Inc., Gaithersburg, USA) was added to each of the wells of the microplate. Plates were allowed to incubate at room temperature for 30 min. Plates were read at 405 nm using a plate reader. The mean optical densities (ODs) of uninfected hamster serum at each dilution and standard deviations were calculated. Positive binding results for infected hamsters were characterized ODs by being three standard deviations greater than the mean ODs of the uninfected hamster serum at the corresponding dilution. The endpoint titer of a given sample was calculated as the highest dilution giving a positive binding result.

### Quantitative real-time PCR assay and cytokine analysis

RNA was extracted from whole blood following the protocol for Viral RNA Mini kit (QIAGEN, Limburg, Netherlands). cDNA was synthesized using 0.5 µg total RNA following the protocol for RT2 First Strand Kit (QIAGEN, Limburg, Netherlands). Quantitative real-time PCR for cytokine expression was conducted following the protocol for TaqMan Gene Expression Master Mix (Applied Biosystems, California, USA). Primers and probes targeting hamster-specific cytokine genes (IL-2, IL-4, IL-6, IL-10, IP-10, iNOS, β2M, TGF-β, p27, VEGF, TNF-α and IFN-γ), as well as the RPL-18 internal reference gene, were developed previously^43^ and used at final concentrations of 900-nM and 250-nM, respectively. The final reaction volume was 20 µL and the thermal cycling conditions for the qRT-PCR assay were as follows: initial denaturation at 95 °C for 3 min followed by 40 cycles of denaturation at 95 °C for 20 s and annealing/extension at 60 °C for 35 s. The Comparative CT method described previously^44^ was used to quantify relative cytokine expression.

### Statistical analysis

One-way ANOVA followed by Dunnett’s multiple comparisons tests was performed using GraphPad Prism version 8.0.0 for Windows (GraphPad Software, San Diego California USA, www.graphpad.com). Means and standard errors are reported.

## Results

### Susceptibility of STAT2^*−/−*^ hamsters to lethal disease via multiple routes of CCHFV infection

**Figure 1:**
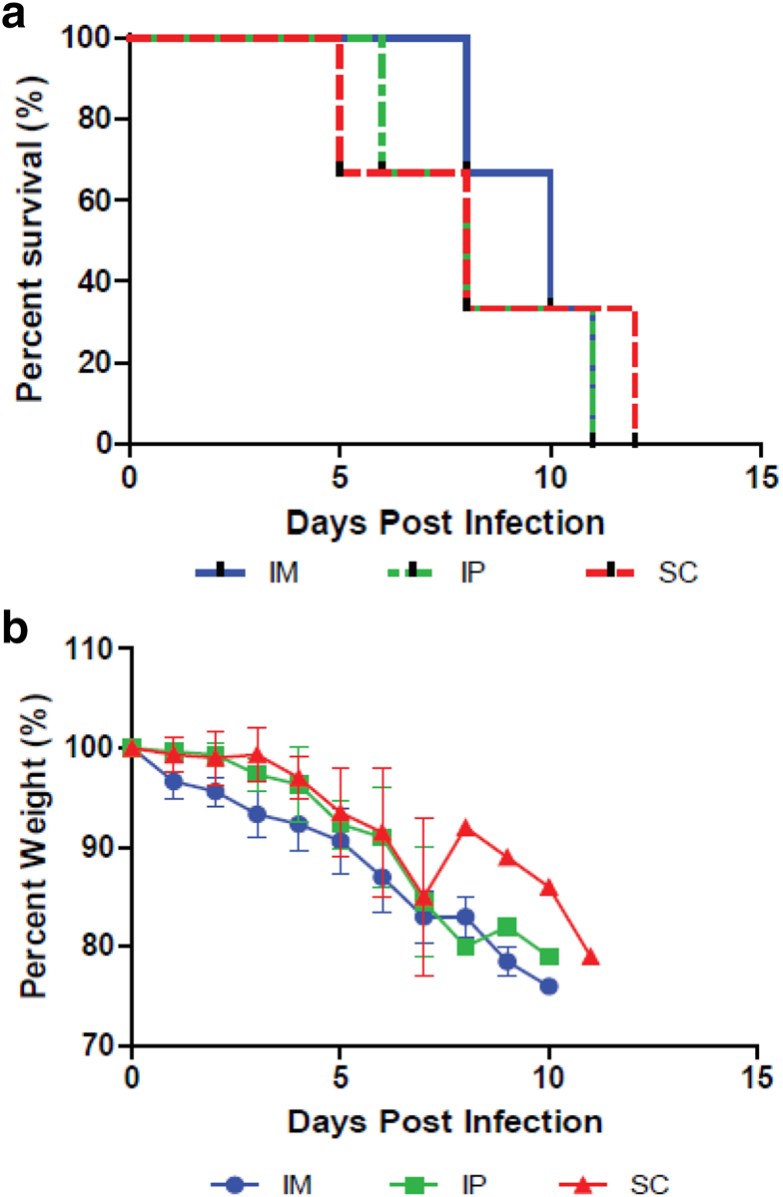
Assessment of multiple routes of infection of CCHFV in *STAT2*^*−/−*^ hamsters. Hamsters were infected with 10,000 TCID_50_ IbAr10200 CCHFV by either the intraperitoneal, intramuscular or subcutaneous route of infection (n = 3). Percent survival (**a**) and percent body weight (**b**) was determined throughout infection was calculated. One-way ANOVA followed by Dunnett’s multiple comparisons tests was performed using GraphPad Prism version 8.0.0 for Windows (GraphPad Software, San Diego California USA, www.graphpad.com). Means and standard errors are reported.

It has been well established that adult, immunocompetent animals including Syrian hamsters are refractory or not susceptible to clinical disease manifestations following inoculation with CCHFV^45,46^ (Rosenke, Safronetz, Feldmann unpublished data). To establish an alternative small animal model of CCHFV infection, we assessed the susceptibility of *STAT2*^*−/−*^ hamsters to 10,000 TCID_50_ CCHFV inoculation via intraperitoneal, intramuscular, and subcutaneous route. *STAT2*^*−/−*^ hamsters demonstrated disease and mortality by all routes of infection tested. Animals infected by the subcutaneous or intraperitoneal routes had a mean time to death of 8.3 dpi and animals infected intramuscularly had a mean time to death of 9.6 dpi (Fig. 1). All animals were euthanized by 12 dpi upon observing the predetermined humane endpoints.

### Disease course and systemic distribution of CCHFV in STAT2^*−/−*^ hamsters

**Figure 2:**
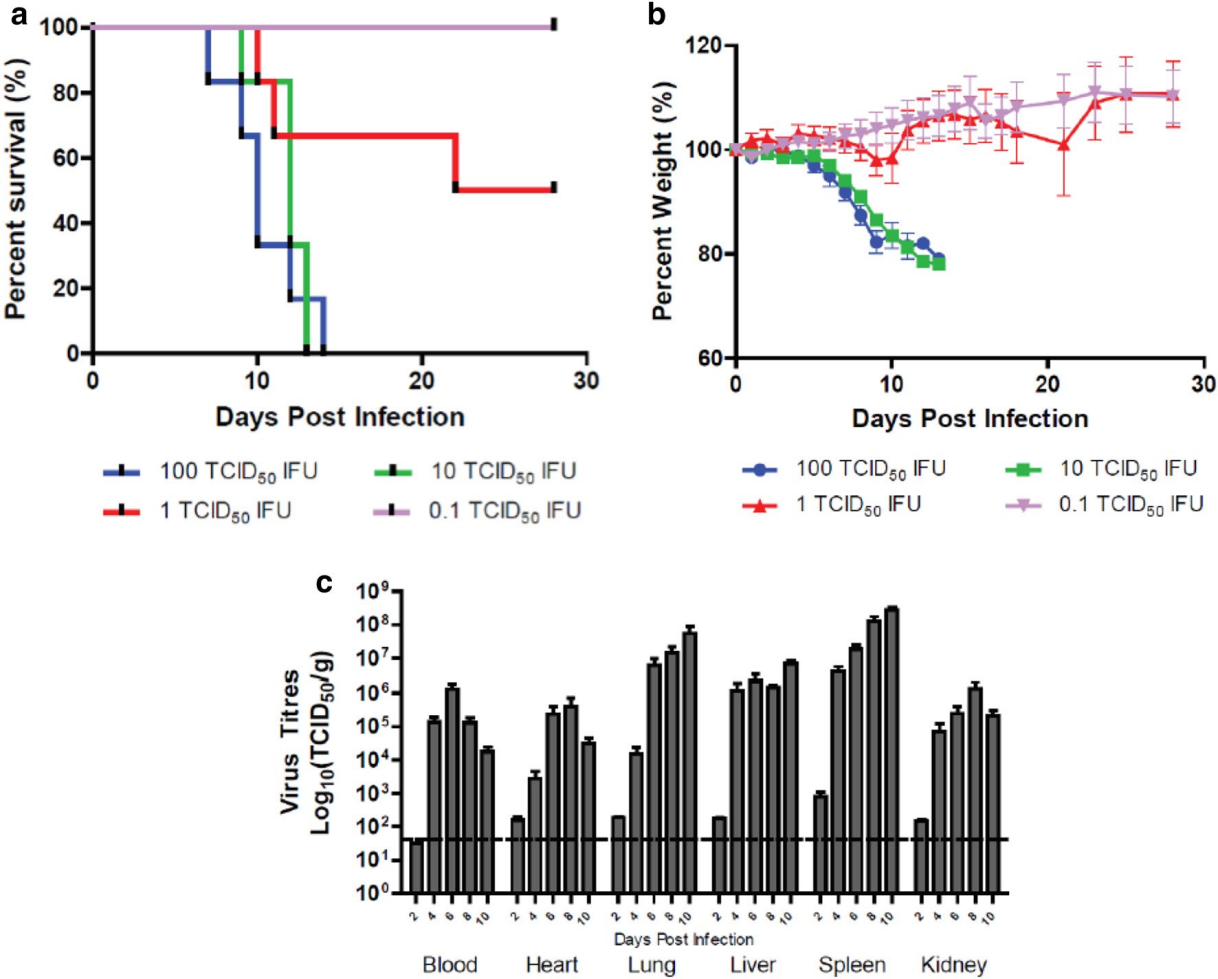
LD_50_ determination and virus distribution of CCHFV in *STAT2*^*−/−*^ hamsters. Hamsters were subcutaneously infected with various doses of IbAr10200 CCHFV (n = 6). Percent survival (**a**) and percent body weight (**b**) was determined. In a follow-up study, hamsters were subcutaneously infected with 100 LD_50_ (100 TCID_50_) of CCHFV, humanely euthanized at 2, 4, 6, 8 and 10 dpi (n = 5/time point) and cardiac blood, heart, lungs, liver, spleen and kidneys were harvested and tittered for the presence of CCHFV on SW13 cells (**c**). One-way ANOVA followed by Dunnett’s multiple comparisons tests was performed using GraphPad Prism version 8.0.0 for Windows (GraphPad Software, San Diego California USA, www.graphpad.com). Means and standard errors are reported.

Since subcutaneous inoculation resulted in lethal infection and more closely mimics the natural route of infection by intradermal tick bite, this route was used for all subsequent experiments. We inoculated *STAT2*^*−/−*^ hamsters with ten-fold serial dilutions of virus and monitored for weight loss and clinical signs of disease to establish the median (50%) lethal dose (LD_50_) of CCHFV administered subcutaneously and characterize the time course and signs of disease progression. The CCHFV strain IbAr10200 had an LD_50_ of 1 TCID_50_ IFU, with 50% of animals succumbing to infection between 10 and 22 dpi (Fig. 2a). This is similar to those described or inferred from previous reports using immunodeficient murine models, often with different viral titration methods and inoculation routes ^31–34^ though hamsters demonstrated a prolonged disease course. Hamsters typically died between 9 and 13 dpi when inoculated with 10 and 100 TCID_50_ IFU, doses which consistently killed 100% of animals (Fig. 2a). Animals with fatal disease typically showed significant weight loss (> 20%) (Fig. 2b). Additionally euthanasia decisions were based on a combination of other clinical manifestations, some or all of which were present days prior to euthanasia (hunched posture, ruffled and dull coat condition, aggressive behaviour, severe dehydration, lethargy, laboured breathing, disorientation and jerky movements) or immediately perimortem (righting reflex issues and/or hind limb paralysis, approximately 70% of animals; visible ocular, anal, or petechial hemorrhaging, approximately 40%).

To characterize viral replication and tropism in the *STAT2*^*−/−*^ hamster model of CCHFV infection, hamsters were infected subcutaneously with 100 LD_50_ (100 TCID_50_) of CCHFV and serially sampled on 2, 4, 6, 8 and 10 dpi for temporal analysis of viral kinetics. Blood and organs were harvested and analyzed for the presence of virus (Fig. 2c). Virus was detected in all samples tested but was found at the highest levels in the lung, liver and spleen. Virus replication peaked in the blood at 8 dpi and then declined on 10 dpi, the termination point of the study (Fig. 2c). The levels of virus found in the heart and kidney appeared to follow a similar trend as with the blood, where a decline at later time points was observed (Fig. 2c). However, viral replication continued to increase in the spleen, lungs, and to a lesser degree in the liver (Fig. 2c), despite the decreasing replication elsewhere.

### Pathological findings in STAT2^*−/−*^ hamsters infected with CCHFV

**Figure 3:**
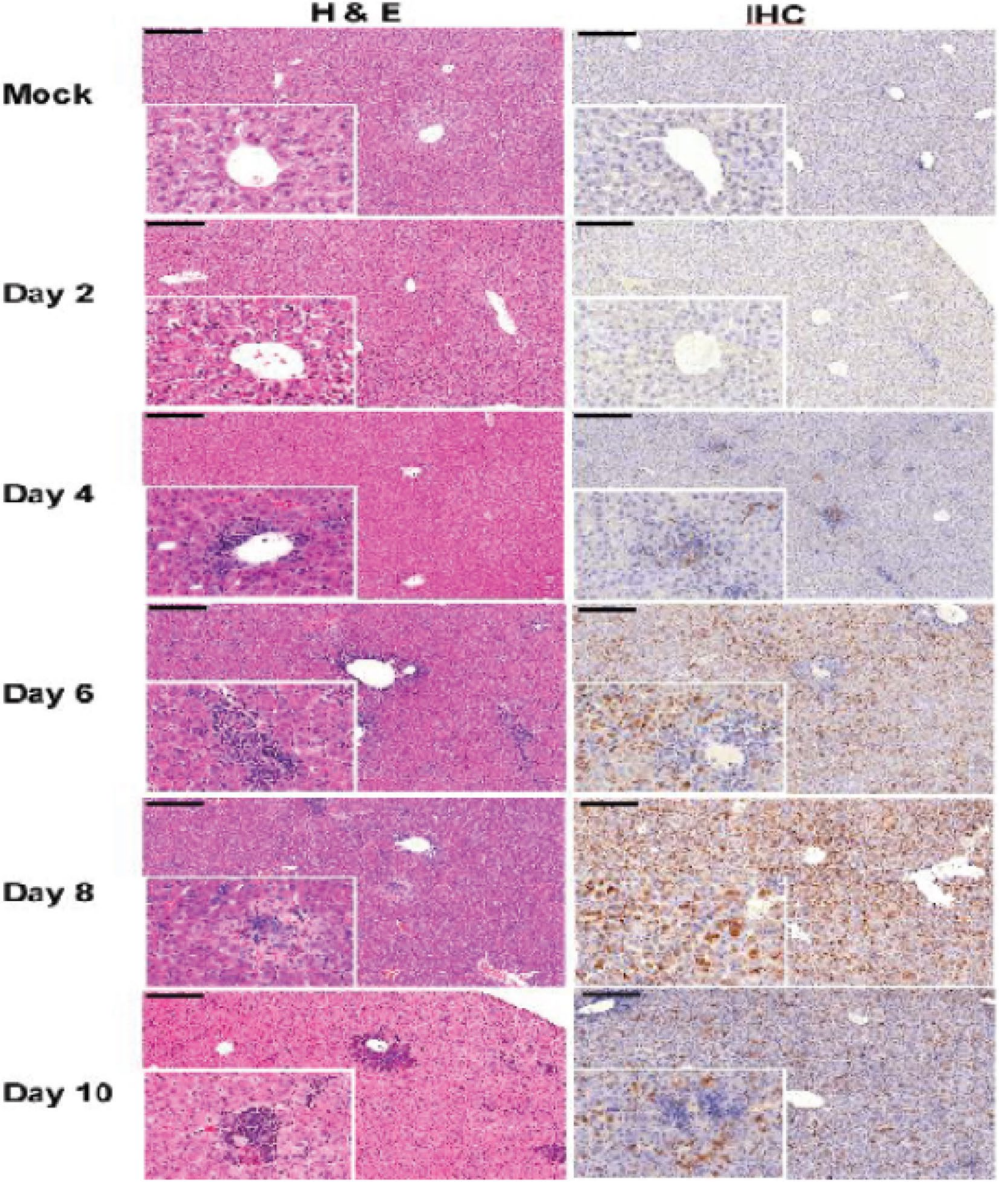
Liver histopathology and immunohistochemistry evaluation of *STAT2*^*−/−*^ hamsters infected with CCHFV. Hamsters were subcutaneously infected with 100 LD_50_ (100 TCID_50_) of IbAr10200 CCHFV. Animals were humanely euthanized at 2, 4, 6, 8 and 10 dpi (n = 5/time point) with liver samples harvested at each time point. Tissues were stained with hematoxylin and eosin or with a mouse antibody against the CCHFV NP antigen. Pathologic changes and viral staining were apparent by day 4 post infection and increased over time. The liver displayed foci of hepatocellular necrosis and inflammation by 4 dpi which was extensive throughout the tissue by day 6 after infection. Bar = 200 µm.

**Figure 4:**
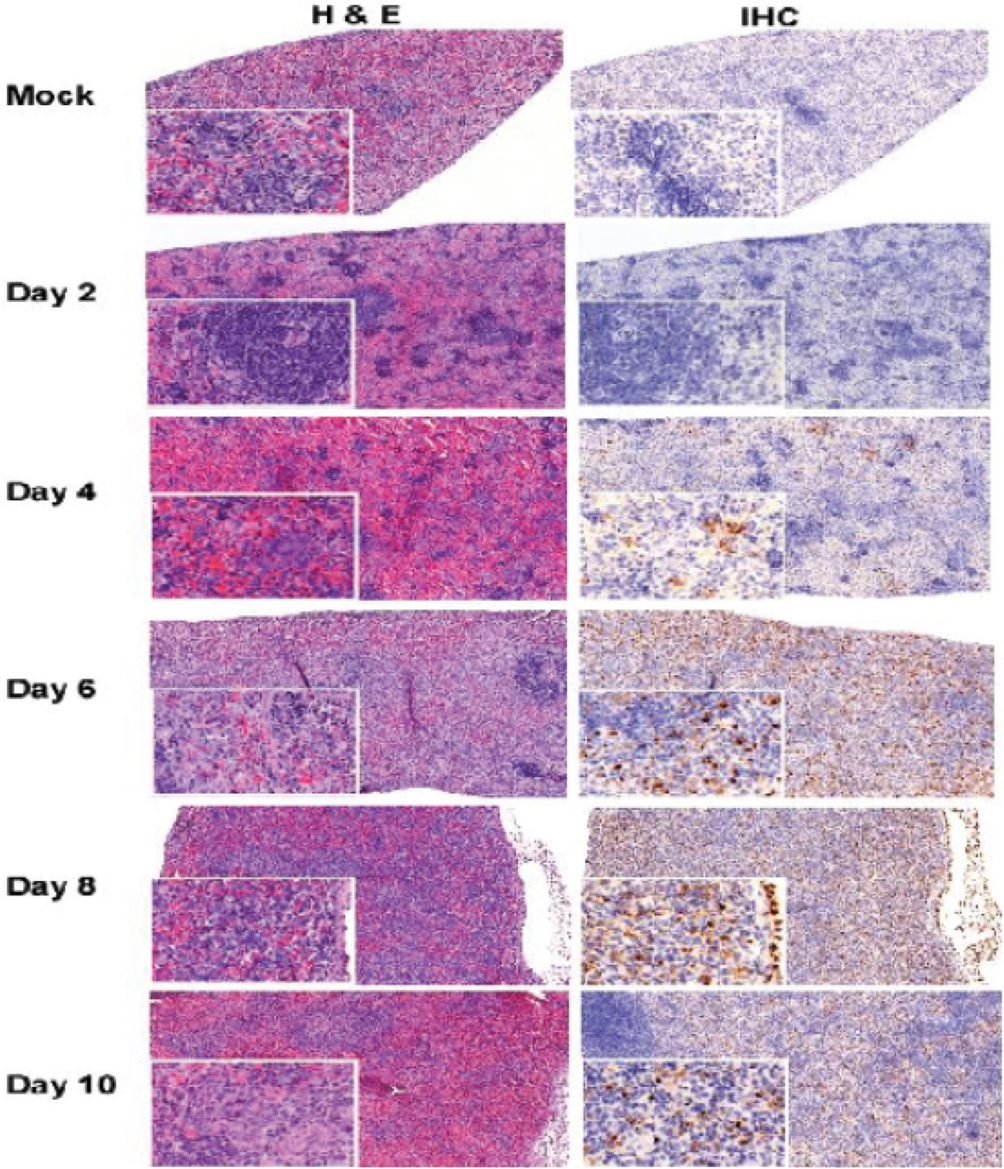
Spleen histopathology and immunohistochemistry evaluation of *STAT2*^*−/−*^ hamsters infected with CCHFV. Hamsters were subcutaneously infected with 100 LD_50_ (100 TCID_50_) of IbAr10200 CCHFV. Animals were humanely euthanized at 2, 4, 6, 8 and 10 dpi (n = 5/time point) with spleen samples harvested at each time point. Tissues were stained with hematoxylin and eosin or with a mouse antibody against the CCHFV NP antigen. Pathologic changes and viral staining were apparent by day 4 post infection and increased over time. Marked loss of white pulp and virus staining was evident in spleen tissue by day 4 after infection. Bar = 200 µm.

**Figure 5:**
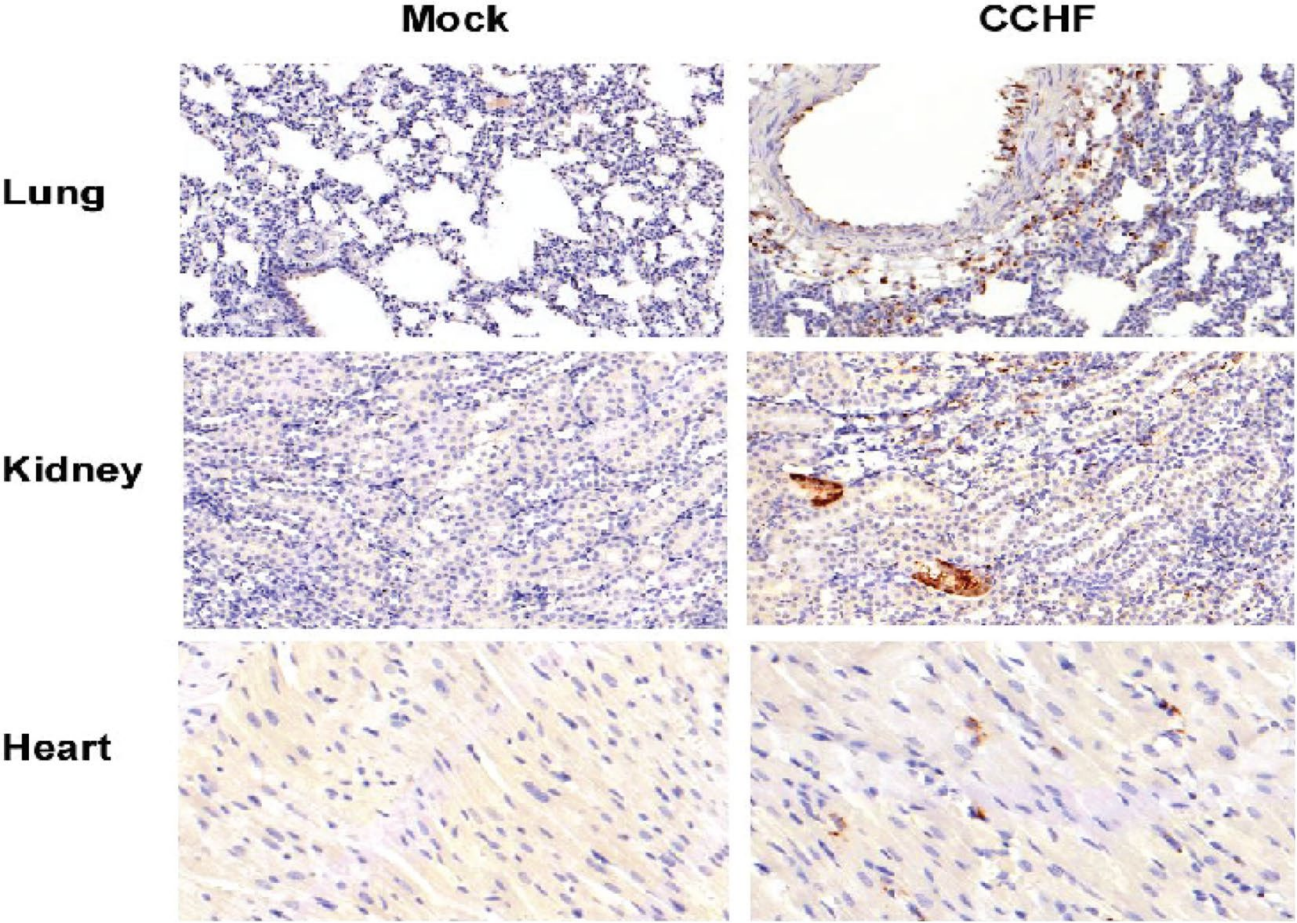
Immunohistochemistry evaluation of *STAT2*^*−/−*^ hamsters infected with CCHFV. Hamsters were subcutaneously infected with 100 LD_50_ (100 TCID_50_) of IbAr10200 CCHFV. Animals were humanely euthanized at 2, 4, 6, 8 and 10 dpi (n = 5/time point) and lungs, kidneys and heart were harvested. Tissues were stained with hematoxylin and eosin or with a mouse antibody against the CCHFV NP antigen. Shown are representative images from animals 8 dpi, approximately two days after initial antigen staining appeared in these tissues and a time corresponding to peak antigen staining. No virus-induced pathology was noted in the kidney, heart or lungs of either mock- or infected hamsters. Immunoreactive cells for CCHFV antigen were widespread in lung tissues, particularly the epithelial and endothelial cells and alveolar septal cells, from day 6 onwards. Small foci or individual cells stained in kidney and heart tissues. Bar = 50 µm.

Gross pathological changes were observed in organs of STAT2^*−/−*^ hamsters infected with 100 LD_50_ (100 TCID_50_) of CCHFV during disease progression. Compared to control animals, enlarged spleens were first observed at 4 dpi, the appearance of pale livers and kidneys started at 6 dpi, and the development of hemorrhages on the skin and surface of the liver were noticed between 8 and 10 dpi. Histopathology and IHC analysis were performed on the liver, spleen, lungs, kidney and heart tissues of infected and mock-infected animals collected at each time-point. Tissues from all mock-infected animals exhibited normal pathology and no viral antigen was detected at any time-point (Figs. 3, 4, 5). Histologic examination showed the liver and spleen to be the primary organs of infection with pathologic changes apparent beginning on 4 dpi and increasing over the course of the disease (Figs. 3, 4). Liver contained multiple foci of hepatocellular necrosis and inflammation throughout the tissue by 6 dpi (Fig. 3). By 8 dpi loss of hepatic architecture, necrosis and areas of intense inflammatory infiltration were extensive (Fig. 3). Staining for CCHFV antigen was not detected at 2 dpi but by 4 dpi small numbers of hepatocytes, either alone or in small groups, had cytoplasmic immunoreactivity in at least 50% of hamsters (Fig. 3). At least 40% of hepatocytes stained positive for CCHFV antigen by 8 dpi. Loss of lymphocytes was observed in splenic white pulp in addition to some infiltration of neutrophils starting at 4 dpi when staining of viral antigen was apparent within foci of cells (Fig. 4). By 6 dpi virus immunoreactivity was prominent throughout the tissue. No discernable virus induced pathology was noted in the kidney, heart and lungs of either mock- or infected hamsters (Fig. 5). However, immunoreactive cells were widespread in lung tissues, particularly the epithelial and endothelial cells and alveolar septal cells, from 6 dpi onwards (Fig. 5). Individual or small foci of cells stained positive for CCHFV antigen in kidney and heart tissues (Fig. 5).

### Serological findings in STAT2^*−/−*^ hamsters infected with CCHFV

**Figure 6:**
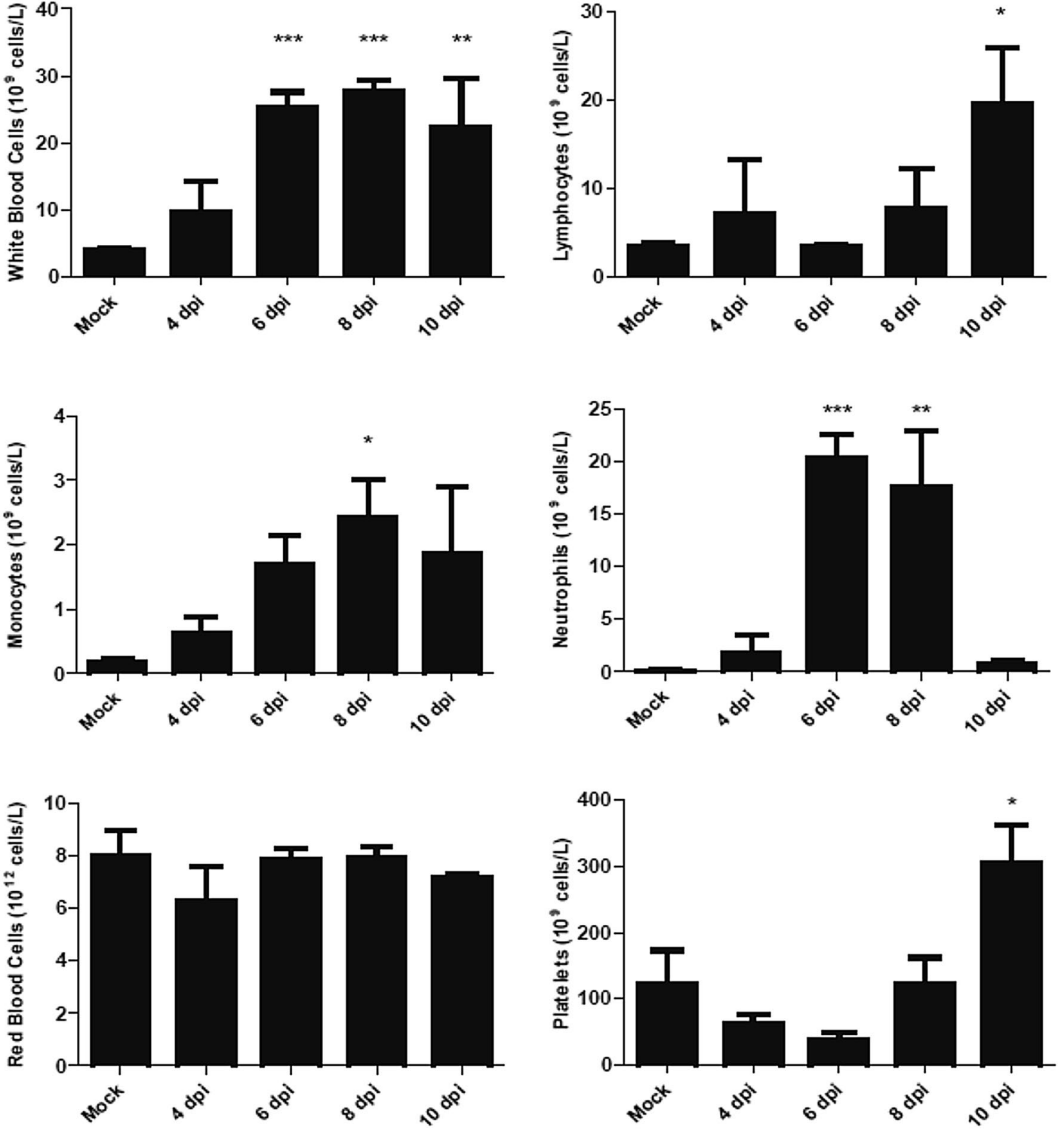
Hematology assessment in CCHFV-infected *STAT2*^*−/−*^ hamsters. Hamsters were subcutaneously infected with 100 LD_50_ (100 TCID_50_) of IbAr10200 CCHFV. Animals were humanely euthanized at 2, 4, 6, 8 and 10 dpi (n = 5/time point) and whole blood was collected and total cell counts were determined. One-way ANOVA followed by Dunnett’s multiple comparisons tests was performed using GraphPad Prism version 8.0.0 for Windows (GraphPad Software, San Diego California USA, www.graphpad.com). Means and standard errors are reported. *P < 0.05, **P < 0.01, ***P < 0.001, compared to mock-infected animals.

During disease progression, significant increases in circulating white blood cells were observed in CCHFV-infected animals compared to control animals at 6, 8 and 10 dpi, with a spike in lymphocytes observed at the termination time point, 10 dpi (Fig. 6). Monocytes demonstrated an upward trend over time but the only statistically significant increase compared to control animals occurred at 8 dpi (Fig. 6). The presence of circulating neutrophils in the blood spiked at 6 and 8 dpi and then returned to baseline at 10 dpi when the experiment was terminated, suggesting possible early release of neutrophils from the bone marrow, exhaustion of neutrophil production, or stress-related demargination causing the rapid influx of neutrophils into the blood and then the rapid infiltration into tissues (Fig. 6). The latter supports our pathology data, which first detected neutrophils in the spleen at 4 dpi. Red blood cells, and hemoglobin levels were unaffected by infection. Platelet counts, however, were affected by infection and demonstrated a complex pattern, with a steadily decreasing trend between 0 and 6 dpi, followed by a significant elevation by 10 dpi (Fig. 6).

**Figure 7:**
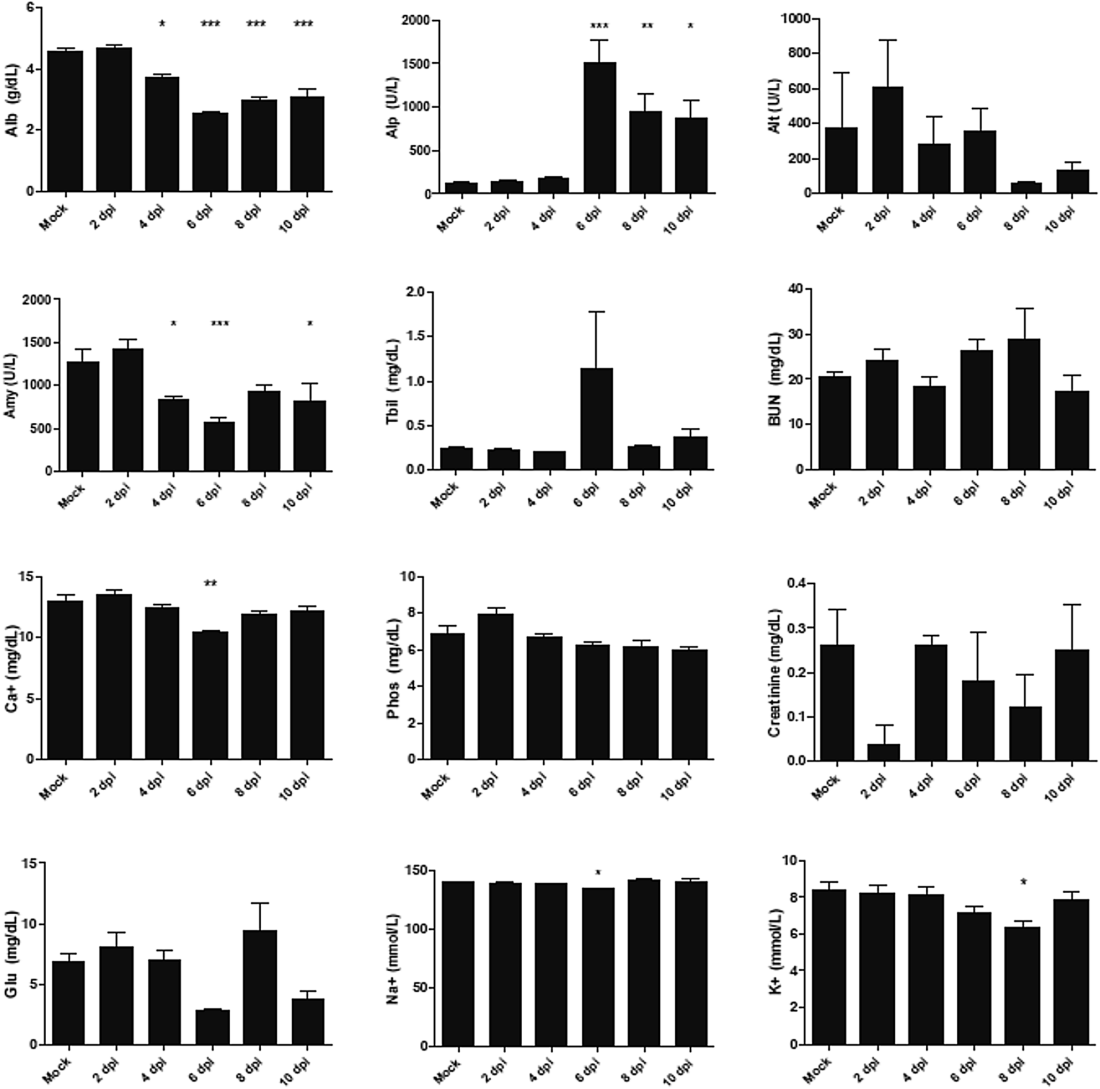
Evaluation of clinical blood chemistries of *STAT2*^*−/−*^ hamsters infected with CCHFV. Hamsters were subcutaneously infected with 100 LD_50_ (100 TCID_50_) of IbAr10200 CCHFV. Animals were humanely euthanized at 2, 4, 6, 8 and 10 dpi (n = 5/time point) and serum was collected and various biochemical markers were assessed. One-way ANOVA followed by Dunnett’s multiple comparisons tests was performed using GraphPad Prism version 8.0.0 for Windows (GraphPad Software, San Diego California USA, www.graphpad.com). Means and standard errors are reported. *P < 0.05, **P < 0.01, ***P < 0.001, compared to mock-infected animals.

Blood chemistries were assessed throughout CCHFV infection in STAT2^*−/−*^ hamsters. Albumin (Alb) and total protein (TP) decreased over the course of disease; however, globulin levels began to increase at later time points (Fig. 7), suggesting that protein synthesis remained functional and the decrease in Alb and TP was due to losses from the digestive or renal systems. While not statistically significant, ALT levels were elevated at early time points during infection indicative of early signs of liver damage (Fig. 7). ALP levels were significantly elevated at later time points, suggesting widespread tissue dysfunction given its released from multiple sources: liver, heart and muscle tissue (Fig. 7). This finding is supported by the pathology findings observed, describing late-stage liver cirrhosis due to infection with CCHFV in the *STAT2*^*−/−*^ hamsters. BUN, creatinine and K + levels make it unclear whether renal function is affected, even though there was active virus replication, visual changes in kidney tissue colouration, and foci of CCHFV-specific immunoreactivity in the tissues of the kidneys.

**Figure 8:**
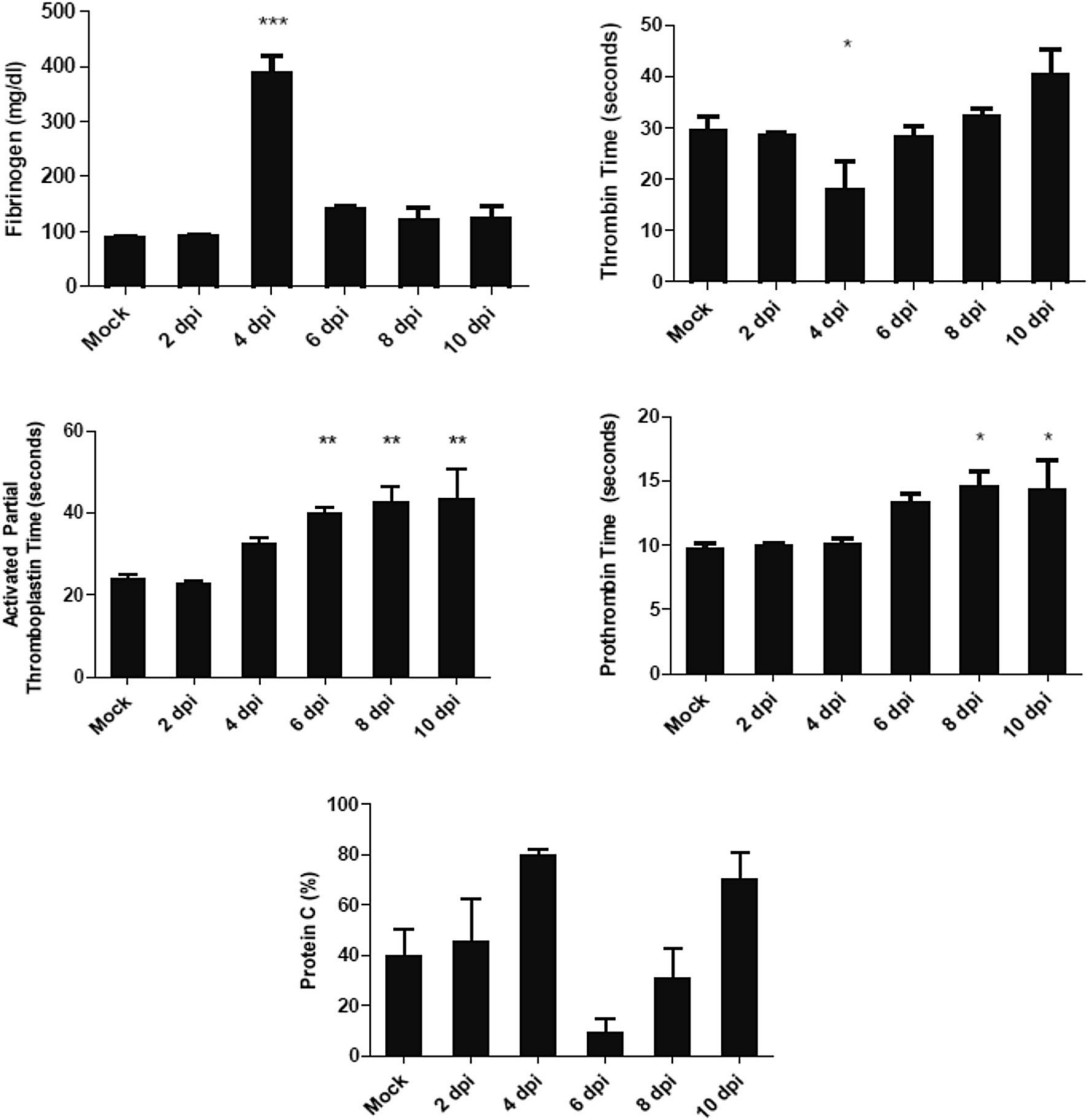
Observing of coagulation parameters following CCHFV infection in *STAT2*^*−/−*^ hamsters. Hamsters were subcutaneously infected with 100 LD_50_ (100 TCID_50_) of IbAr10200 CCHFV. Animals were humanely euthanized at 2, 4, 6, 8 and 10 dpi (n = 5/time point). Fibrinogen, thrombin time, activated partial thromboplastin time, prothrombin time and protein C percentages were measured. One-way ANOVA followed by Dunnett’s multiple comparisons tests was performed using GraphPad Prism version 8.0.0 for Windows (GraphPad Software, San Diego California USA, www.graphpad.com). Means and standard errors are reported. *P < 0.05, **P < 0.01, ***P < 0.001, compared to mock-infected animals.

Blood coagulation factors were assessed from fresh plasma at the time of sample collection. Increasing clotting times were observed for prothrombin and activated partial thromboplastin (Fig. 8). Thrombin times remained stable except for a transient decline in clotting time at 4 dpi and then returned to baseline levels. Interestingly, this decline at 4 dpi correlated with a significant increase in fibrinogen concentrations while at all other time points, baseline levels were maintained. There were no statistically significant changes in the percentage of protein C, although the pattern of changes is noteworthy. Levels increased from baseline to 4 dpi, became exhausted at 6 dpi, and then continued to increase until the time of termination (Fig. 8). These results could be indicative of disseminated intravascular coagulation. The ability to replenish the protein levels also indicate that protein synthesis remains intact, while decreased levels are caused by protein consumption or loses.

### Immunological findings in STAT2^*−/−*^ hamsters infected with CCHFV

**Figure 9:**
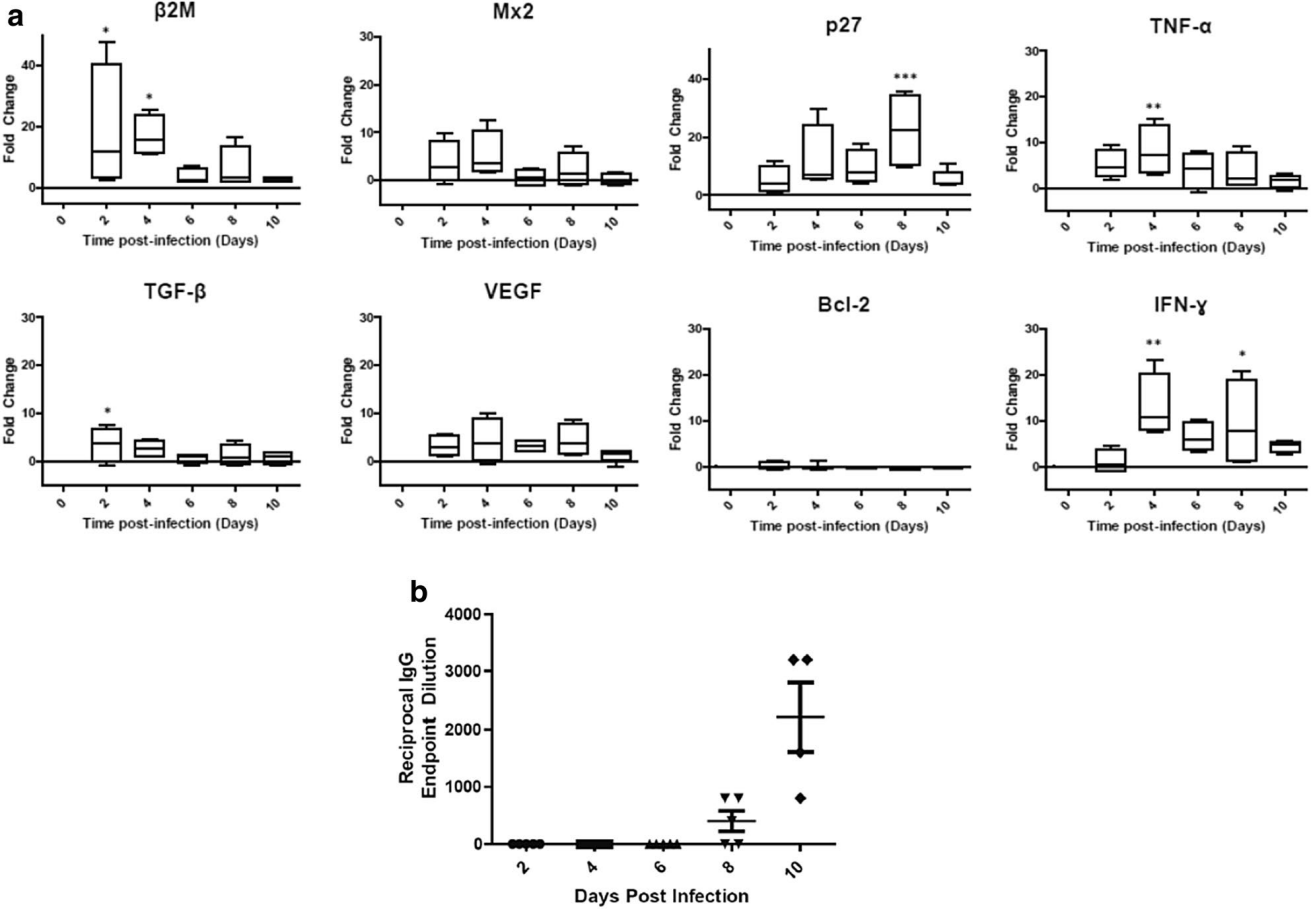
Immune responses following CCHFV infection in *STAT2*^*−/−*^ hamsters. Hamsters were subcutaneously infected with 100 LD_50_ (100 TCID_50_) of IbAr10200 CCHFV. Animals were humanely euthanized at 2, 4, 6, 8 and 10 dpi (n = 5/time point). Fold changes of β2M, Mx2, TGF-β, VEGF, p27, TNF-α, Bcl-2, and IFN-γ were calculated (**a**). One-way ANOVA followed by Dunnett’s multiple comparisons tests was performed using GraphPad Prism version 8.0.0 for Windows (GraphPad Software, San Diego California USA, www.graphpad.com). Means and standard errors are reported. *P < 0.05, **P < 0.01, ***P < 0.001, compared to mock-infected animals. Reciprocal endpoint dilutions (**b**) were expressed as the reciprocal of the lowest serum dilution giving a positive binding result compared with the negative control. The mean and standard error is shown for each time point.

Since immunological reagents for hamsters are limited, RNA was extracted from whole blood and various cytokine/chemokine transcript levels were assessed by quantitative RT-PCR. We tested 14 different cytokines and chemokines and were unsuccessful at detecting mRNA transcripts or were unable to calculate a fold change due to undetectable baseline levels in the mock-infected animals for six of the cytokines (IL2, IL4, IL6, IL10, IP10, and iNOS). However, we were able to detect and calculate fold changes for 8 of the cytokines we assessed (Fig. 9a). Bcl2, Mx2 and VEGF did not show any significant changes in their transcript levels; however, β2M, TGFβ, p27, TNFα and IFNγ transcripts were upregulated at various time points during infection (Fig. 9a). The presence of β2M, TGFβ,and TNFα transcripts were significantly upregulated in the blood at early time points during infection, usually between 2 and 4 dpi; while, p27 transcripts were increased at later time points during infection at 8 dpi, and finally IFNγ transcripts were significantly upregulated at both 4 and 8 dpi (Fig. 9a). These results suggest that some innate immune responses mounted upon infection remained functional in the immunodeficient *STAT2*^*−/−*^ hamster model. Since cytokine activation was limited we wanted to determine whether this model was also capable of producing an adaptive antibody response. Attempts at generating an ELISA to detect anti-CCHFV IgM antibodies were unsuccessful. However, we were able to develop an anti-CCHFV IgG assay and serum collected from infected animals was assayed. Anti-CCHFV nucleoprotein-specific IgG antibodies were detected in 60% of hamsters at 8 dpi and 100% of hamsters had detectable levels at 10 dpi (Fig. 9b). These data demonstrate that while some aspects of the immune response are impaired by the knock out of the *STAT2* gene, these animals were still capable of developing antibodies targeting CCHFV nucleoprotein in response to infection.

### Application in antiviral evaluation

**Figure 10:**
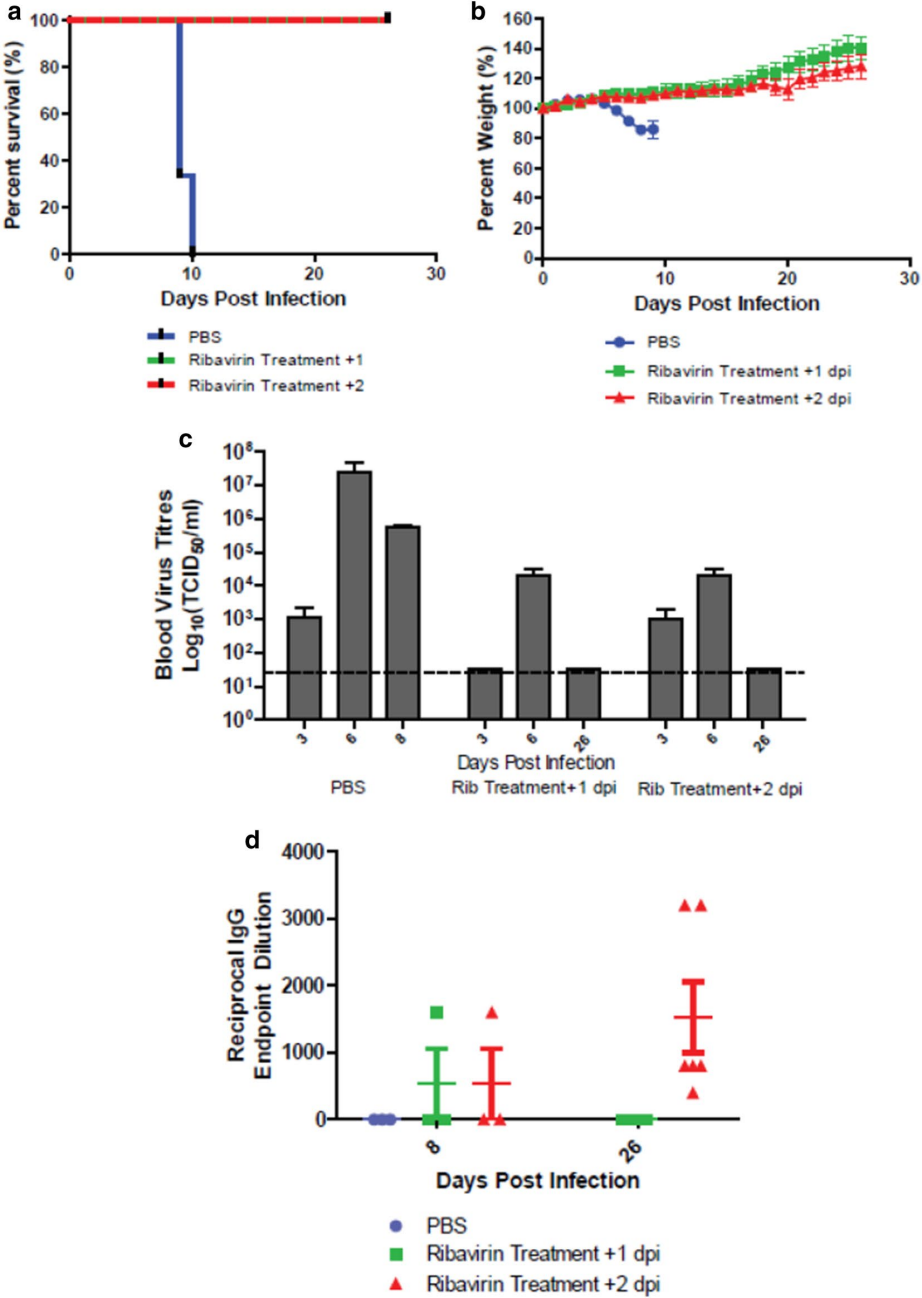
Efficacy determination of Ribavirin treatment on *STAT2*^*−/−*^ hamsters infected with CCHFV. Hamsters were subcutaneously infected with 100 LD_50_ (100 TCID_50_) of IbAr10200 CCHFV. Animals received two daily 100 mg/kg doses of Ribavirin beginning on 1 (n = 9)or 2 dpi (n-9), the daily dose was then reduced to 75 mg/kg and administered for a further 12 days. Percent survival (**a**) and weight loss (**b**) was monitored daily (n = 6). Trial bleeds were collected from hamsters on day 3 and 6 (n = 3) and terminal bleeds were conducted at the termination of the experiment (n = 6). Viremia was determined by TCID_50_ (**c**). One-way ANOVA followed by Dunnett’s multiple comparisons tests was performed using GraphPad Prism version 8.0.0 for Windows (GraphPad Software, San Diego California USA, www.graphpad.com). Means and standard errors are reported. (**d**) Three animals were euthanized on 8 dpi and the remaining six animals were euthanized at the termination of the study on 26 dpi. The presence of CCHFV-specific IgG antibodies were detected from serum. Reciprocal endpoint dilutions were expressed as the reciprocal of the lowest serum dilution giving a positive binding result compared with the negative control. The mean and standard error is shown for each time point.

The efficacy of ribavirin for the treatment of CCHFV infection remains the subject of much debate in both human cases and existing small animal models. To evaluate the utility of the STAT2^*−/−*^ hamster model to test therapeutics against CCHFV, hamsters were infected with 100 LD_50_ of CCHFV (100 TCID_50_) and treated with ribavirin beginning on either 1 or 2 dpi. Hamsters were treated with a loading dose of 100 mg/kg/day of ribavirin for two days and then the dose of ribavirin was dropped to 75 mg/kg/day for 12 additional days. All ribavirin-treated animals survived infection, showed no signs of weight loss, and were terminated on 26 dpi at the completion of the study, while all PBS treated animals demonstrated rapid weight loss and were humanely euthanized by 10 dpi (Fig. 10a,b). Viremia was detected in treated animals at 3 and/or 6 dpi but not at 26 dpi (Fig. 10c). When the presence of anti-CCHFV IgG antibodies were assessed, 2 of the 6 ribavirin-treated animals had detectable levels at 8 dpi and 100% of animals that began treatment at 2 dpi still had detectable levels at 26 dpi, while animals that began treatment 1 dpi had no detectable antibody levels (Fig. 10d).

## Discussion

CCHFV is a zoonotic virus that causes disease in humans and in severe cases leads to death. With the growing understanding of prognostic factors that predict mortality in human CCHFV cases, the ideal animal model for the evaluation of therapeutic agents should exhibit similar signs and laboratory findings in order to better predict drug efficacy in humans. In this study, we established the first lethal hamster model for the study of CCHFV disease and pathogenesis. Lethally infected *STAT2*^*−/−*^ hamsters exhibit extended time to death (9–13 dpi) compared with the *STAT1*^*−/−*^^31^ and *IFNAR*^*−/−*^^32–34^ mouse models of CCHFV which succumb to illness at 2–5 dpi, but shorter than the Hu-NSG-SGM3 humanized mouse model (13–23 dpi)^36^. CCHFV infection in the *STAT2*^*−/−*^ hamsters consists of an incubation period between 1 and 5 dpi where there are no clinical signs of infection, followed by the manifestation of disease on day 6 post-infection which continues until the animals reach their euthanasia endpoints. This finding aligns well with the time course of human fatalities, which includes an incubation time of 1–13 days, followed by a disease state for 5–14 days prior to death^47^.

The *STAT2*^*−/−*^ hamster model for CCHFV infection demonstrated signs of human infection not yet characterized in previous small animal models. To our knowledge, this is the first small animal model for CCHFV in which splenomegaly, petechial rash and orbital bleeding were observed. These findings align with studies identifying petechiae as a prognostic factor in human CCHFV infections^15,18^, as well as a recent study characterizing CCHFV infection in non-human primates^30^. Furthermore, this model also captures clinical signs that are associated with neurologic illness observed in some human cases^48–53^ and characterized in the Hu-NSG-SGM3 humanized mouse model^36^. Future CCHFV studies using the *STAT2*^*−/−*^ hamster model may investigate whether these observations are significant between infected and non-infected animals, as well as surviving and non-surviving animals.

Decreased fibrinogen, prothrombin elongation, and activated partial thromboplastin time are coagulation findings associated with mortality in human cases of CCHFV^14,15,18^. Similarly, the *STAT2*^*−/−*^ hamster model established in this study exhibits a similar dysregulation of both the extrinsic (prothrombin elongation) and intrinsic (activated partial thromboplastin elongation) coagulation pathways upon infection, whereas *IFNAR*^*−/−*^ mice only demonstrate increased clotting times for activated partial thromboplastin and no change in prothrombin clotting times^34^. In contrast, fibrinogen is increased upon infection in this model, similar to findings observed in the IFNAR^*−/−*^ mouse model^34^, but not observed in human cases. This finding also correlated with a decrease in thrombin clotting time, suggesting an abundance of fibrinogen substrate for the production of fibrin by thrombin. Both fibrinogen and thrombin levels returned to baseline levels at 6 dpi and for the duration of infection. This increase in fibrinogen for both the *IFNAR*^*−/−*^ and *STAT2*^*−/−*^ models may be a rodent-specific observation. Alternatively, fibrinogen is a marker of early inflammation; therefore, it is possible we are observing a spurious increase rather than a true rise in coagulopathy. Thrombocytopenia is a significant prognostic factor in human CCHF cases which has also been demonstrated in the *STAT1*^*−/−*^, *IFNAR*^*−/−*^ and humanized mouse models for CCHFV infection. As expected, there was an observed trend of platelet decline in the *STAT2*^*−/−*^ hamsters by 6 dpi, but interestingly peaked again by 10 dpi. To date, it remains controversial whether an increase or decrease in white blood cells (leukocytosis and leukopenia, respectively) is associated with mortality in human CCHF cases. In contrast to the *STAT1*^*−/−*^ mouse model which displays leukopenia, the *STAT2*^*−/−*^ hamster exhibits an increase in white blood cells similar to the Hu-NSG-SGM3 humanized mouse model upon infection. A decline in liver function is a hallmark of hemorrhagic fever viruses^54–59^. The pattern of liver disease was notable in the *STAT2*^*−/−*^ hamsters. We observed a significant amount of viral replication and pathology in the liver in infected hamsters. ALT levels demonstrated relatively little increase compared to the extent of damage seen by pathology. This could be due to an insensitive ALT assay or very rapid progression to liver damage, making the brief ALT rise difficult to observe. Together, this demonstrates the onset of liver failure due to infection with CCHFV and mimics the findings observed in humans and in the *IFNAR*^*−/−*^ and *STAT1*^*−/−*^ mouse models^14–16,18,31,34^.

As expected, the *STAT2*^*−/−*^ hamsters exhibited impairment in the Type 1 IFN response, shown by a lack of change in the expression of bcl-2 and absence of iNOS and IP-10 induction upon infection. However, similar to the *STAT1*^*−/−*^ and *IFNAR*^*−/−*^ mice, the *STAT2*^*−/−*^ hamsters developed an increase in TNF-α, a prognostic factor in human CCHF cases^17^. Unfortunately, we were unable to observe any changes in IL-6 and IL-8, which are also predictive indicators of mortality in humans. At 2 dpi, we observed a significant increase in expression of β2M, a plasma protein subunit of MHC class I and other class I-like molecules (CD1, Qa) in humans. β2M is upregulated and shed by lymphocytes upon stimulation with IFN-γ, which occurred in the *STAT2*^*−/−*^ hamsters upon infection^60^. The increase in β2M could suggest T-cell activation, which is elicited by a variety of viral infections^61^ and remains functional in this immunodeficient model of infection. We also assessed the ability of this model to generate CCHFV-specific antibodies. Presence of anti-CCHFV IgG antibodies was first detected at 8 dpi, a late stage of infection. After treatment with ribavirin we were able to detect the presence of CCHFV-specific antibodies at 8 dpi in 33% of animals and then 100% of animals that began treatment 2 dpi had detectable levels of CCHFV-specific antibodies at 26 dpi. The presences of antibodies in the other models were neither assessed nor detected^31,32,34,36^. The ability of the *STAT2*^*−/−*^ hamster model to generate antibodies in response to infection is encouraging and would suggest that this platform could be used to not only test for potential therapeutic agents but also vaccine candidates.

Finally, we showed that ribavirin could prevent mortality of *STAT2*^*−/−*^ hamsters infected with CCHFV, demonstrating the model’s ability to be used for the evaluation of promising CCHFV therapeutic candidates. The efficacy of ribavirin against CCHFV mortality demonstrated in this study aligns with the results of a similar evaluation in which 100% of *STAT1*^*−/−*^ mice were protected from CCHFV challenge when ribavirin treatment was initiated at 1 dpi^31^. In contrast, ribavirin was ineffective in IFNAR^*−/−*^ mice, demonstrating only 14% survival when administered on 0 dpi^33^. The latter study implemented a different CCHFV isolate and may explain the change in outcome. However, a recent study using the same *IFNAR*^*−/−*^ mouse platform demonstrated similar results, a 0% and a 33% survival rate when IbAr10200 and Hoti strains were employed, respectively, suggesting that ribavirin may not be a suitable candidate for the treatment of CCHFV infection or that its success may be model dependent^62^. The ambiguous efficacy of ribavirin in preventing fatality in animal models mirrors that in humans, which shows benefit in some studies but ineffectiveness in others^5,11,63–68^. However, these findings may provide insights into host factors that work synergistically or antagonistically with ribavirin treatment. Furthermore, the heterogeneity in ribavirin efficacy in humans may be in part due to relatively late administration of the treatment. This animal model could be used to define a window of efficacy for ribavirin, which could better inform clinical practice.

The *STAT2*^*−/−*^ hamster model of CCHFV infection builds upon findings from previous rodent models and demonstrates a comprehensive disease profile mimicking many aspects of human infection. The model was able to recapitulate a variety of clinical signs observed in humans, with the visualization of petechial rash and orbital hemorrhaging being unique characteristics that makes it distinctive from other animal models. Additionally, the presence of altered liver enzymes, coagulation markers and changes in cell blood counts are also representative of the disease profile in humans. This lethal model demonstrates viremia and widespread virus replication throughout a variety of organ systems. Furthermore, the ability of this model to generate an antibody response and the ability to respond to antiviral treatment suggest that the *STAT2*^*−/−*^ hamster would be a suitable model for the evaluation of promising antiviral drug and vaccine candidates.
